# A Bayesian decision approach to classify crime scene observations in sharp-force fatalities: a study on suicide vs. homicide scenarios

**DOI:** 10.1007/s00414-026-03756-7

**Published:** 2026-03-16

**Authors:** Franco Taroni, Silvia Bozza, Guido Pelletti, Arianna Giorgetti, Paolo Fais, Marco Irmici, Susi Pelotti

**Affiliations:** 1 School of Criminal Justice, The University of Lausanne, Lausanne, Switzerland; 2 Department of Economics, Ca’ Foscari University of Venice, Venice, Italy; 3 Department of Medical and Surgical Sciences, University of Bologna, Bologna, Italy

**Keywords:** Bayesian approach, Bayesian networks, Bayesian logistic regression, Decision theory, Forensic medicine, Probabilistic graphical models, Sharp-force fatalities, Suicide, Homicide

## Abstract

A Bayesian decision approach is proposed to address complex discrimination problems in forensic medicine. From a judicial perspective, the accurate assessment of cases of violent death as a suicide or a homicide is of undeniable importance for criminal prosecution, guiding decisions on the depth of forensic investigation and the requirement for additional analytical procedures. Inaccurate decisions can have severe consequences; therefore, a normative perspective is introduced to provide a framework through which current practice can be articulated. This study represents a proof of principle based on a dataset of 51 cases (24 suicides and 27 homicides). A Bayesian logistic regression model is implemented to assign a probability value to the event that a questioned scenario belongs to a given population (e.g., suicide or homicide). However, the computation of such probability and the subsequent decision-making process remain a demanding task. Probabilistic graphical models, specifically Bayesian Networks (BNs), offer a suitable and user-friendly framework to assist forensic pathologists in providing statistically sound expert opinions. The implementation of the proposed BN facilitates the application of this approach in routine forensic practice.

## Suicide vs homicide: a medico-legal current problem

Distinguishing between homicides and suicides based on medical and circumstantial data represents a fundamental challenge in sharp-force forensic pathology. Even when the cause of death is clearly identified, the manner of death is crucial for criminal prosecution. Such discrimination would significantly assist magistrates during investigations, guiding decisions on whether to deepen forensic investigation and require additional analytical procedures. In sharp-force fatalities, variables commonly used to differentiate between homicide and suicide are described in classic [1] and modern [2] textbooks of forensic medicine. The evaluation of the manner of death often relies on a combination of multiple factors, such as the nature and pattern of lethal wounds, the presence of characteristic injuries like defensive wounds or hesitation marks, the condition of the victim’s clothing, the age of the deceased, and the circumstances surrounding the death scene [3, 4].

In 1998, Karlsson [5, 6] was the first to address the need for developing a predictive model based on commonly observed variables in forensic investigations. The aim was to inform–rather than replace–the diagnostic decision-making process of forensic pathologists. However, in the subsequent years, few retrospective studies and reviews were published with the goal of identifying the most frequently observed features in homicide and suicide cases. Among these studies, only a few focused on comparative assessments of the relevant variables observed during post-mortem examination and crime scene investigation, which would allow for the comparison of frequencies [7–9].

In a recent comparative study [10], existing forensic literature on sharp-force fatalities was reviewed, specifically focusing on studies that reported the manner of death and the frequency of traditionally assessed characteristics. This resulted in a database of 173 suicides and 354 homicides. The probative value of each characteristic, independently assessed and expressed as a likelihood ratio, was reported and calculated for a series of sharp-force deaths with a known manner of death. The victim’s gender and the number of injuries showed similar frequencies in both homicides and suicides. However, the presence of psychiatric illness, post-mortem alcohol detection, and the type and location of the sharp object used were strongly associated with the manner of death. Injuries to the thorax and lower limbs were significantly more frequent in homicides, while injuries to the crook of the arm and wrist were more often observed in suicides.

In Italy^1^, as in other European countries^2^, sharp-force homicides represent by far the most frequent manner of death amongst homicides. The expert witnesses often find themselves testifying on the cause and manner of death, with the necessity of discriminating when a differential diagnosis between homicides and suicides is required. The aim of this paper is to propose a Bayesian decision approach for discriminating between suicide and homicide in sharp-force fatalities. Indeed, in both medical and legal contexts, diagnosis is not an end in itself, but rather a means to assist subsequent decisions regarding medical treatment or judicial verdicts. The proposed solution allows questioned scenarios to be assigned to a given population (suicide or homicide) by including values for consequences of inaccurate decisions in the assignment process through the use of normative decision analysis. As in other fields of forensic science, the use of a Bayesian perspective for evidence evaluation, inference and decision-making has been strongly encouraged in forensic pathology as well (see, e.g., [11–13]). Such an approach is highly recommended due to its logical desiderata [14, 15].

To facilitate both probabilistic reasoning and the often tedious inferential computations, we propose the use of a probabilistic graphical model, specifically a Bayesian network (BN). Bayesian networks are widely discussed and applied in judicial and forensic literature as both inferential and decisional tools (see, e.g., [14, 16–19]).

The remainder of this paper is structured as follows. Section “Bayesian decision framework” briefly describes the Bayesian way of reasoning for inference and decision-making. In Section “Material and methods”, we detail the available data and the proposed statistical model. Section “Results” presents the results obtained from applying the statistical model to the available dataset. Section “Bayesian networks for discriminating the manner of death” translates the proposed probabilistic approach into a user-friendly Bayesian network, reproducing the results from Section “Results” and demonstrating its capability to handle the scenarios of interest without requiring tedious mathematical calculations. Section “A decisional perspective: Bayesian decision networks” proposes a normative decisional extension aimed at providing a coherent justification for rationally assigning cases of violent death to a given population. Finally, Section “Discussion and conclusion” concludes the paper.

## Bayesian decision framework

In Bayesian analysis, all available information is utilized to manage the uncertainty inherent in inferential problems. As new information is obtained, it is combined with prior knowledge to update the scientist’s state of information, forming the basis for statistical procedures. At any given point in time, the scientist’s state of information about uncertain events of interest can be represented by a set of probabilities. The role of probability theory in inductive logic is to show how probabilities of unobserved events (e.g., the population of origin of a set of findings) are to be modified in the light of new data as to represent all available knowledge about the case at hand. This process is centered on Bayes’ theorem, which represents the fundamental rule for conducting such reasoning in compliance with the requirement of rationality [20].

Let us denote by $H_1$ and $H_2$ the hypotheses of interest that can be formulated as follows:

- $H_1$: the manner of death is suicide;
- $H_2$: the manner of death is homicide.

Let us denote by *E* the available information, comprising both circumstantial data (e.g., the place where the body was found) and physical findings from the forensic examination (e.g., the characteristics of sharp-force injuries). We denote by *I* the background information (i.e., contextual knowledge, such as witness testimony). A Bayesian probabilistic model can be implemented to infer the posterior probability $\Pr (H_i\mid E,I)$ of the competing propositions, *i=1,2*. The formal derivation of these probabilities is detailed in Section “Bayesian logistic regression”.

The Bayesian paradigm can be extended to encompass the perspective of decision making. A decision problem arises when there is a list of possible courses of action (also called decisions) and there is uncertainty about some events (e.g. hypotheses) called states of nature and consequently about the consequences that may arise from each of the possible decisions. In our context, a decision corresponds to the expert’s formal opinion regarding the manner of death to be submitted to the legal authority, while the consequences refer to the potential impact of this assessment on the judicial proceedings. For example, providing an opinion of suicide for what is actually a homicide could lead to the premature dismissal of a criminal investigation.

Bayesian decision theory offers a transparent framework for a rational and coherent justification of one’s choices [21]. More specifically, decision theory specifies how to make rational (and formally justified) choices between courses of action under uncertainty. Let $D=\{d_1, d_2\}$ denotes the decision space, where $d_1$ represents the decision to report a case of violent death as a suicide; vice versa, decision $d_2$ is specified as the decision to report a case of violent death as a homicide. Decision $d_{1}$ is correct if hypothesis $H_{1}$ is true (i.e., the manner of death is suicide); similarly, decision $d_{2}$ is correct if hypothesis $H_{2}$ is true (i.e., the manner of death is homicide). Conversely, decision $d_{1(2)}$ is not correct if hypothesis $H_{1(2)}$ is not true.

A decision problem can be expressed as a decision matrix (Table 1), where $C_{ij}$ denotes the consequence of taking decision $d_i$ when hypothesis $H_j$ is true (*i, j = 1, 2*). For instance, $C_{12}$ represents the consequence of providing an assessment of suicide ($d_1$) when the manner of death is actually a homicide ($H_2$). To quantify the gravity of these outcomes, a loss function *L(· )* is introduced. In a two-action decision problem such as the one at hand, it is standard to use a $0-l_i$ loss function. In this framework, the loss is zero for correct assessments: $C_{11}$ and $C_{22}$ represent the scenarios where the expert’s opinion matches the true state of nature (i.e., a suicide is reported as such, or a homicide is reported as such), leading to $L(C_{11})=0$ and $L(C_{22})=0$. Conversely, a positive loss is assigned to erroneous decisions to reflect their relative gravity. Specifically, $l_1=L(C_{12})$ represents the loss incurred when a homicide ($H_2$) is incorrectly reported as a suicide ($d_1$); this value quantifies the serious consequence of potentially halting a criminal investigation and leaving a perpetrator at large. Similarly, $l_2=L(C_{21})$ represents the loss for reporting a suicide ($H_1$) as a homicide ($d_2$); this reflects the burden of an unjustified investigation, which may involve unnecessary judicial costs. This allows us to weigh the relative cost of erroneous decisions.

**Table 1:** Decision table for a discrimination problem. The table illustrates the consequences $C_{ij}$ (columns 2–3), the general losses $L(C_{ij})$ (columns 4–5), and the specific $0-l_i$ loss function (columns 6–7) where $l_i$ represents the loss for an incorrect decision $d_i$

Decisions	Hypotheses		Hypotheses		Hypotheses	
	$H_{1}$	$H_{2}$	$H_{1}$	$H_{2}$	$H_{1}$	$H_{2}$
	Consequences		Loss function		$0-l_i$ Loss function	
$d_{1}$: suicide	$C_{11}$	$C_{12}$	$L(C_{11})$	$L(C_{12})$	0	$l_1$
$d_{2}$: homicide	$C_{21}$	$C_{22}$	$L(C_{21})$	$L(C_{22})$	$l_2$	0

The losses $l_1$ and $l_2$ may be equal, whenever the adverse decision outcomes are considered equally undesirable; otherwise, the magnitude of each loss will represent the undesirability of each specific outcome resulting in an asymmetric loss function. The relative undesirability of the outcomes of available decisions can be expressed by their *expected losses* $\textrm{EL}(\cdot )$, which are computed as:

1 $$\begin{aligned} \textrm{EL}(d_1)= & \textrm{L}(C_{11}) \Pr (H_1 \mid E,I) + \textrm{L}(C_{12}) \Pr (H_2 \mid E,I)\end{aligned}$$

2 $$\begin{aligned} \textrm{EL}(d_2)= & \textrm{L}(C_{21}) \Pr (H_1 \mid E,I) + \textrm{L}(C_{22}) \Pr (H_2 \mid E,I) \end{aligned}$$

Given the $0-l_i$ loss function, the expected losses in Eq. 1 and 2 simplify to:

$$\begin{aligned} \textrm{EL}(d_1)= & \underbrace{\textrm{L}(C_{11})}_{0} \Pr (H_1 \mid E,I) + \underbrace{\textrm{L}(C_{12})}_{l_1} \Pr (H_2 \mid E,I)=l_1\Pr (H_2\mid E,I)\\ \textrm{EL}(d_2)= & \underbrace{\textrm{L}(C_{21})}_{l_2} \Pr (H_1 \mid E,I) + \underbrace{\textrm{L}(C_{22})}_0 \Pr (H_2 \mid E,I)=l_2\Pr (H_{1}\mid E,I). \end{aligned}$$

Essentially, the expected loss quantifies the *overall risk* of each available assessment (decision) by combining the probability of an incorrect conclusion with its associated gravity. It is calculated by weighing the probability of making an error (i.e., reporting a suicide when it is a homicide, or vice versa) by the gravity (loss) of that specific error. Consequently, a decision might be discouraged not only if it is likely to be wrong, but also if the potential mistake–though unlikely–would lead to an unacceptably high social or legal cost.

The formal Bayesian decision criterion is to take decision $d_1$ (decide in favour of hypothesis $H_1$) if the expected loss of decision $d_1$ ($\textrm{EL}(d_1))$ is smaller than the expected loss of decision $d_2$ ($\textrm{EL}(d_2))$ , that is if:

3 $$\begin{aligned} \underbrace{l_1\Pr (H_2\mid E,I)}_{\textrm{EL}(d_1)}< & \underbrace{l_2\Pr (H_1\mid E,I)}_{\textrm{EL}(d_2)}. \end{aligned}$$

Rearranging terms in Equation (3), the Bayes decision criterion states that decision $d_1$ is optimal whenever the ratio of the expected losses is smaller than 1

4 $$\begin{aligned} \frac{\textrm{EL}(d_1)}{\textrm{EL}(d_2)}=\frac{l1}{l2}\times \frac{\Pr (H_2\mid E,I)}{\Pr (H_1\mid E,I)}<1. \end{aligned}$$

This approach constitutes a coherent decision-making procedure, as it minimizes the probability of error [21].

When a symmetric loss function is deemed appropriate ($l_1 = l_2$, i.e., adverse decision outcomes are considered equally undesirable), the optimal criterion becomes to take decision $d_1$ whenever the posterior odds against $H_1$ are smaller than 1, that is whenever the probability of homicide is smaller than the probability of suicide.

However, a symmetric loss function might not always be the most appropriate choice in this context. In an investigative phase of the criminal procedure, for instance, one might argue that falsely classifying a case of violent death as a suicide should be regarded more severely than falsely classifying it as a homicide. This is because an actual homicide would be declared a suicide, which would halt the investigation and prevent the identification of the perpetrator. The Bayesian decision framework is particularly valuable in this regard, as it can be easily adapted to accommodate such asymmetric loss functions, allowing the optimality threshold to be tuned according to the relative gravity of the different types of errors. This aspect will be discussed in Section “A decisional perspective: Bayesian decision networks”.

Specialised statistical, legal and forensic literature introduced formal decision theoretic developments for a variety of decision-making problems arising in legal contexts, such as making decisions about ultimate issues, forensic identification (individualization), sampling and classification, among several others^3^.

## Material and methods

### The available data

A total of $m_1 = 24$ suicide scenarios and $m_0 = 27$ homicide has been collected through a retrospective analysis of autopsy cases referred to the Unit of Legal Medicine, University of Bologna. Sharp force fatalities were included in the study when all collected data, both autopsy findings and circumstantial information derived from police investigations, were available. Given the retrospective design and the inclusion of cases that had reached a final judicial decision, the analysis was limited to fatalities with an unequivocal manner of death.

For each sharp force fatality, the following variables were collected:

- Demographic variables: gender and age of the victim.
- Place where the body was found: home, indoor, or outdoor. We distinguished between ‘home’ and ‘indoor’, defining home as the victim’s own residence.
- Body position: prone, seated, or supine.
- Suicide note: present or absent.
- Weapon type: household knife (e.g., kitchen knife, utility/tool knife), other knife (e.g., hunting knife, survival knife, dive knife, pocket knife), other sharp objects (e.g., axe, razor blade, scissors, metal fragments, glass splinters), or not found.
- Weapon location relative to the body: near the body or away from the body, based on immediate proximity. The weapon was classified as near the body when recovered in a position compatible with dropping after self-stabbing (in the cases included in this study, within1 m from the body).
- Distribution of sharp force injuries: presence/absence of sharp force injuries in the body regions reported in Table 2.
- Distribution of other injuries: presence/absence of additional injuries (ecchymoses, bruises, lacerations) in the body regions reported in Table 2.
- Toxicology: post-mortem detection of illicit drugs, alcohol, or pharmaceuticals (benzodiazepines, antidepressants, antipsychotics) in blood (yes/no).
- Psychiatric comorbidities: psychiatric disorders diagnosed during life by a physician (yes/no).
- History of psychoactive substance use: documented history (yes/no).
- Previous suicide attempts: yes/no.

For each of the included cases, all aforementioned variables were available for analysis, with the exception of psychiatric comorbidities, which were missing in four cases. A description is provided in Table 2.

**Table 2:** Description of available variables

Type of variables	Name	Outcome
Demographic data	Gender	*F*, *M*
	Age	*numeric*
Circumstantial data	Place where the body was found	*Home, Indoor, Outdoor*
	Body position	*Prone, Seated, Supine*
	Suicidal notes	*No, Yes*
	Weapon type	*Household knife, Not found*
		*Other knife, Others*
	Weapon place	*Away from the body,*
		*Near the body*
Sharp-force injuries	Sharp Head	*numeric* ; *No, Yes*
(body region)	Sharp Neck	*numeric* ; *No, Yes*
	Sharp Torax	*numeric* ; *No, Yes*
	Sharp Abdomen	*numeric* ; *No, Yes*
	Sharp Back	*numeric* ; *No, Yes*
	Sharp Pelvis	*numeric* ; *No, Yes*
	Sharp Foreharm (anterior)	*numeric* ; *No, Yes*
	Sharp Upper limbs	*numeric* ; *No, Yes*
	Sharp Hands	*numeric* ; *No, Yes*
	Sharp Lower limbs	*numeric* ; *No, Yes*
Other injuries	Head	*numeric* ; *No, Yes*
(body region)	Neck	*numeric* ; *No, Yes*
	Torax	*numeric* ; *No, Yes*
	Abdomen	*numeric* ; *No, Yes*
	Back	*numeric* ; *No, Yes*
	Pelvis	*numeric* ; *No, Yes*
	Foreharm (anterior)	*numeric* ; *No, Yes*
	Upper limbs	*numeric* ; *No, Yes*
	Hands	*numeric* ; *No, Yes*
	Lower limbs	*numeric* ; *No, Yes*
Toxicology	Illicit drugs	*No, Yes*
	Pharmaceuticals	*No, Yes*
	Alcohol	*No, Yes*
Other anamnestic data	Psychiatric comorbidities	*No, Yes*
	Psychoactive substance use history	*No, Yes*
	Past suicidal attempts	*No, Yes*

Among these, qualitative variables ‘Place where the body was found’ (P), ‘Weapon type’ (W), ‘Sharp back’ (S) and ‘Psychiatric comorbidities’ (Pc) where selected on the basis of their ability to provide a probability pointing versus the correct manner of death (i.e., a probability of suicide greater (smaller) than 0.5 when the analysed case had been classified as suicide (homicide). The conditional distributions of the manner of death given the selected variables are displayed in Table 3. These distributions highlight how the probability of suicide versus homicide shifts significantly depending on the specific state of each finding. This provides empirical support for the inclusion of these variables in the model, as they possess clear discriminatory power.

**Table 3:** Conditional distributions of the manner of death (Suicide or Homicide) given the selected variables

	Place			Weapon place		Sharp back		Psychiatric	
Manner	Home	Indoor	Outdoor	Away	Near	No	Yes	No	Yes
Suicide	0.57	0.76	0.15	0.09	0.76	0.57	0.00	0.28	0.89
Homicide	0.43	0.24	0.85	0.91	0.24	0.43	1.00	0.72	0.11

### Bayesian logistic regression

Bayesian logistic regression is a statistical approach that models the relationship between a binary outcome variable and a set of explanatory variables, using the framework of Bayesian inference.

A binary response variable *y* is introduced to characterise the manner of death, where 0 stands for homicide and 1 stands for suicide. Explanatory variables are denoted by *x*. The conditional distribution of the response variable *y* given the state $x_{1i}$ of a specific variable $x_1$, is a Bernoulli, $y_i\sim B(1,p_i)$, that is

5 $$\begin{aligned} {\Pr (y_i=1 \mid x_1=x_{1i}) = p_i.} \end{aligned}$$

Standard linear regression models express $p_i$ as a linear combination of explanatory variables, such as $p_i=\beta _0+\beta _1 x_{1i}$. However, this is problematic whenever the response variable is binary, as it does not guarantee that probability values assigned to $p_i$ remain in the interval (0,1). To overcome this, Generalized Linear Models (GLMs) are used, as they accommodate non-linear dependence structures. A link function is introduced to map the linear predictor to the (0,1) interval. In a logistic regression model, this link function is the *logit transform*

6 $$\begin{aligned} {\text {logit}(p_i)=\log \left( \frac{p_i}{1-p_i}\right) }=\beta _0+\beta _1 x_{1i}. \end{aligned}$$

Consequently, the probability of the binary response *y* is formalized as the following non-linear combination of explanatory variables:

7 $$\begin{aligned} {p_i = \frac{\exp (\beta _0 + \beta _1 x_{1i})}{1 + \exp (\beta _0 + \beta _1 x_{1i})}.} \end{aligned}$$

The available dataset includes several categorical variables. To incorporate these qualitative observations into the model, they are transformed into dummy variables. Each dummy variable consists of 1s for cases belonging to the category of interest and 0*s* for the others. The category for which no dummy variable is created is called the *reference category* and the regression coefficients are interpreted relative to this baseline.

Consider, for example, the variable P (‘Place where the body was found’), which has three categories: *Home*, *Indoor*, *Outdoor*. To include this variable in the model, two dummy variables are created ($x_1$ and $x_2$). We set $x_1=1$ for *Indoor* cases and 0 otherwise. Similarly, we set $x_2=1$ for *Outdoor* cases and 0 otherwise. The *Home* category serves as reference, as shown in Table 4.

**Table 4:** Example of dummy variable coding for the categorical variable ‘Place’. The state *Home* serves as the reference category

Manner of death	*y*	P (Place)	$x_1$ (Indoor)	$x_2$ (Outdoor)
Suicide	1	*Home*	0	0
Suicide	1	*Indoor*	1	0
*⋮ *	*⋮ *	*⋮ *	*⋮ *	*⋮ *
Homicide	0	*Outdoor*	0	1
*⋮ *	*⋮ *	*⋮ *	*⋮ *	*⋮ *

Since all other retained variables are dichotomous, one dummy variable is created for each. Specifically, the reference categories were selected following alphabetical order: *Away from the body* serves as the reference for ‘Weapon place’ (WP), while *No* is the reference for both ‘Sharp back’ (S) and ‘Psychiatric comorbidities’ (Pc). Three dummy variables are therefore created, one for each categorical variable ($x_3$ for ‘Weapon place’, $x_4$ for ‘Sharp back’ and $x_5$ for ‘Psychiatric comorbidities’). Consequently, the full model contains five dummy variables, and the probability $p_i$ in Eq. 7 becomes:

8 $$\begin{aligned} {p_i = \frac{\exp (\beta _0 + \beta _1 x_{1i} + \beta _2 x_{2i} + \beta _3 x_{3i} + \beta _4 x_{4i} + \beta _5 x_{5i})}{1 + \exp (\beta _0 + \beta _1 x_{1i} + \beta _2 x_{2i} + \beta _3 x_{3i} + \beta _4 x_{4i} + \beta _5 x_{5i})}.} \end{aligned}$$

In this setup, each regression coefficient *β * quantifies the effect of a specific finding on the probability of suicide relative to the reference category. For instance, a positive regression coefficient $\beta _1$ for the variable ‘Indoor’ ($x_1$) would indicate that finding a body in an indoor setting increases the probability of suicide compared to cases found at ‘Home’ (the baseline), assuming all other factors remain constant.

To estimate the parameters of this model (i.e., the regression coefficients $\beta _0$–$\beta _5$), a non-informative prior probability distribution was chosen. Due to the non-linear nature of the logistic link function, the posterior probability distribution for the regression coefficients cannot be derived analytically (i.e., there is no closed-form solution). Therefore, numerical integration techniques are required to perform inference. In this study, we rely on Markov Chain Monte Carlo (MCMC) methods, specifically implementing a Metropolis-Hastings algorithm, to obtain samples from the posterior distribution and estimate the model parameters [31].

The estimated regression coefficients and the corresponding dummy variables provide the quantitative basis for the subsequent inferential model. Specifically, this logistic structure will be integrated into a Bayesian network (Section “Bayesian networks for discriminating the manner of death”), that will serve as the numerical engine to calculate the posterior probabilities of the manner of death based on the specific findings of each case (Eq. 8).

## Results

In Tables 5 and 6 there are reported the posterior probabilities of suicide using the Bayesian logistic regression model described in Section “Bayesian logistic regression” with an incremental number of variables. In particular, a first model was fitted using the sole variable ‘Place where the body was found’ (P). Successively, the other selected variables, say ‘Weapon place’ (WP), ‘Sharp back’ (S) and ‘Psychiatric comorbidities’ (Pc) were incrementally added. Additionally, the possibility of interactions between the explanatory variables was investigated. A preliminary logistic regression model including first-order interaction terms was fitted; however, none of the interaction coefficients were found to be significant. Therefore, a main-effects model was preferred to ensure parsimony and to facilitate the subsequent implementation of the Bayesian network.

The suicide probability for each case in the dataset was calculated using a leave-one-out procedure. In this approach, a single case is selected, and all remaining cases are used as background data to fit the Bayesian logistic regression model. Performances of the implemented model offer satisfactory results for both cases where the manner of death is suicide (Table 5) and where the manner of death is homicide (Table 6). The correct classification rates achieve satisfactory values. Regarding suicides, only two cases (S6 and S11) show a probability of suicide lower than 0.5, which would lead to a misclassification as homicide; this results in a correct classification rate of 0.92. Similarly, for homicides, only two cases (H4 and H15) present a probability of suicide greater than 0.5, suggesting an incorrect classification as suicide; this corresponds to a correct classification rate of 0.91. This last rate was calculated over 23 cases (and not 27), as 4 cases were excluded due to missing data (NA) for the variable *Psychiatric comorbidities*.

**Table 5:** Posterior probability of suicide using variable ‘Place where the body was found’ (column 2); variables ‘Place where the body was found’ and ‘Weapon place’ (column 3); variables ‘Place where the body was found’, ‘Weapon place’ and ‘Sharp back’ (column 4); variables ‘Place where the body was found’, ‘Weapon place’, ‘Sharp back’ and ‘Psychiatric comorbidities’ (column 5). The final line called ‘Probability greater than 0.5’ designs the correct classification rate

Manner of death	P	P+W	P+W+S	P+W+S+Pc
Suicide	0.5434712	0.7637816	0.8268744	1.0000000
Suicide	0.5357160	0.7583983	0.8326757	0.7032319
Suicide	0.5521389	0.7449457	0.8322769	0.6986830
Suicide	0.5370496	0.7569320	0.8371494	1.0000000
Suicide	0.5463339	0.7461395	0.8379810	1.0000000
Suicide	0.0800993	0.0014625	0.0034811	0.1231209
Suicide	0.5460944	0.7492372	0.8316110	1.0000000
Suicide	0.7672635	0.9326222	0.9519968	0.9690390
Suicide	0.5461684	0.7399276	0.8372330	1.0000000
Suicide	0.0871895	0.3070620	0.3675636	0.9861415
Suicide	0.0854141	0.0015803	0.0030113	0.1335618
Suicide	0.7682994	0.9280537	0.9546248	1.0000000
Suicide	0.7657921	0.9305055	0.9551611	0.9664582
Suicide	0.7702636	0.9307927	0.9510532	1.0000000
Suicide	0.7661659	0.9324759	0.9536514	0.9715883
Suicide	0.7647295	0.9329795	0.9553218	0.9669653
Suicide	0.7605260	0.9288190	0.9529306	1.0000000
Suicide	0.7642808	0.9279653	0.9515531	1.0000000
Suicide	0.7610461	0.9312577	0.9555871	1.0000000
Suicide	0.7611718	0.9297699	0.9559315	1.0000000
Suicide	0.7628348	0.9333391	0.9548575	0.9719586
Suicide	0.7648874	0.9314917	0.9540124	0.9720505
Suicide	0.7677958	0.9298449	0.9530431	1.0000000
Suicide	0.5398954	0.7538578	0.8335494	1.0000000
Prob > 0.5	0.875	0.875	0.875	0.92

**Table 6:** Posterior probability of suicide using variable ‘Place where the body was found’ (column 2); variables ‘Place where the body was found’ and ‘Weapon place’ (column 3); variables ‘Place where the body was found’, ‘Weapon place’ and ‘Sharp back’ (column 4); variables ‘Place where the body was found’, ‘Weapon place’, ‘Sharp back’ and ‘Psychiatric comorbidities’ (column 5). The final line called ‘Probability smaller than 0.5’ designs the correct classification rate. No information concerning the presence of ‘Psychiatric comorbidities’ was availables for cases (lines) 22, 23, 25 and 27 (NA)

Manner of death	P	P+W	P+W+S	P+W+S+Pc
Homicide	0.1389558	0.5045938	0.6250651	0.0000000
Homicide	0.1414286	0.0173863	0.0000000	0.0000000
Homicide	0.6204970	0.0876517	0.2074813	0.1238142
Homicide	0.1388218	0.5104925	0.6002006	1.0000000
Homicide	0.1416396	0.5166799	0.5988293	0.0000000
Homicide	0.6280394	0.0833818	0.0000000	0.0000000
Homicide	0.6320675	0.0826494	0.1891623	0.1217288
Homicide	0.1367519	0.0161734	0.0000000	0.0000000
Homicide	0.1361425	0.0169032	0.0000000	0.0000000
Homicide	0.1408021	0.0169098	0.0350043	0.0000000
Homicide	0.1410300	0.0185800	0.0306607	0.0000000
Homicide	0.1370880	0.0178168	0.0331679	0.0000000
Homicide	0.6252081	0.8588446	0.0000000	0.0000000
Homicide	0.1367099	0.0175243	0.0357996	0.0000000
Homicide	0.8357376	0.3208797	0.6482242	1.0000000
Homicide	0.1336226	0.4956513	0.0000000	0.0000000
Homicide	0.1384304	0.0177936	0.0330151	0.0000000
Homicide	0.1392283	0.4984841	0.6175565	0.0000000
Homicide	0.8359611	0.3266675	0.0000000	0.0000000
Homicide	0.1376457	0.0170964	0.0351912	0.0000000
Homicide	0.8337120	0.2963372	0.0000000	0.0000000
Homicide	0.1363602	0.0170998	0.0337427	NA
Homicide	0.1325502	0.0162824	0.0302563	NA
Homicide	0.6278986	0.0845681	0.2022043	0.1348463
Homicide	0.6205740	0.8547162	0.9307941	NA
Homicide	0.1397671	0.0158458	0.0000000	0.0000000
Homicide	0.8354031	0.3002781	0.6660603	NA
Prob < 0.5	0.63	0.81	0.74	0.91

## Bayesian networks for discriminating the manner of death

Forensic literature and practice are becoming more and more accustomed to probabilistic graphical models, such as Bayesian networks (BNs). Originally developed in the field of artificial intelligence, BNs provide a robust framework for applying probability theory to complex inferential problems [32, 33].

Since the late 1980s, BNs have also attracted researchers in forensic science [34, 35], and this tendency has considerably intensified throughout the past decades (see, e.g., [17, 19, 36–41]).

A BN can be defined as a pictorial representation of probabilistic dependencies and influences (represented by arcs) among variables (represented by nodes) relevant to a specific inferential problem. They form the basis for probability propagation algorithms that allow for exact updating of variable probabilities. A directed arc from node *B* to node *A* signifies that variable *B* (the ancestor, or parent node) directly influences variable *A* (the descendant, or child node). Formally, a BN combines graph theory–providing a qualitative structure–with probability theory, which characterizes the strength of the relationships within the model.

The actual state of a variable may not be known with certainty. For example, there may be uncertainty about whether the proposition that the manner of death is suicide is true or not. Within a BN, such a proposition is conceptualized as a Boolean node, where its states represent the truth and falsity of that proposition. The degree of belief held in each of these states is expressed in terms of probabilities. These probabilities are organized in node’s probability tables. The probability table associated to a variable, say *A*, that receives incoming arcs from variables $B_1,...,B_n$ contains conditional probabilities $\Pr (A \mid B_1,...,B_n)$. In contrast, the probability table associated to a variable *A* with no incoming arcs from other variables contains unconditional probabilities *\Pr (A)*^4^.

The core task of a Bayesian network is to process newly acquired information by calculating the conditional probabilities of the states of the nodes of interest (the *hypothesis nodes*) given the observed states of other nodes (the *evidence nodes*). In our context, this involves determining the probability of the manner of death (e.g., ‘suicide’) based on observed findings such as the place where the body was found.

**Fig. 1:**
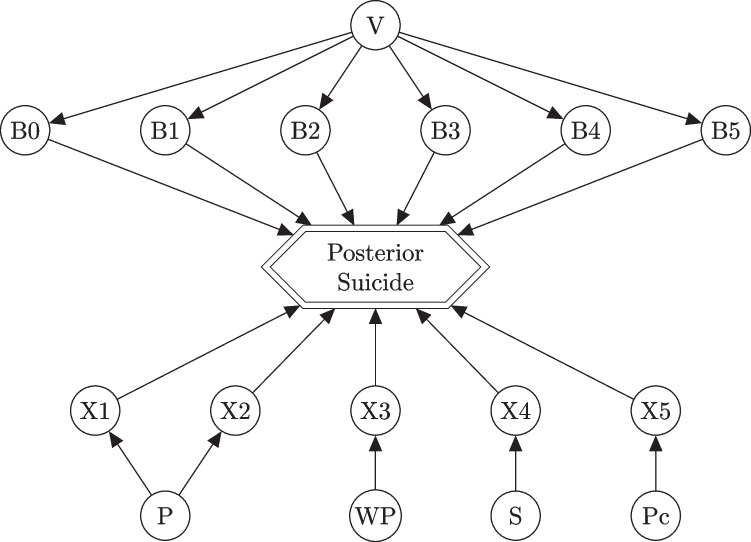
Bayesian network for inference on the manner of death, suicide or homicide, based on a set of observations collected at the crime scene. Node definitions are given in Tables 7, 8 and 9

Figure 1 illustrates the BN developed to describe the scenarios of interest and to produce these probability values for the the suicide hypotheses.

Nodes represents the selected explanatory variables (*V*), the available variables (*P*, *WP*, *S* and *Pc*), the regression coefficients ($\beta _0$–$\beta _5$), and a set of dummy variables ($x_1$–$x_5$) used to encode qualitative data, as detailed in Section “Bayesian logistic regression”. Node descriptions are provided in Tables 7, 8 and 9. It is important to note that the logistic regression model is refitted depending on the number of explanatory variables selected (*V=1, … , 4*). Consequently, the estimated regression coefficients are model-specific and will vary according to the specific subset of variables included in the analysis (as detailed in Table 8, columns 3–4). For instance, when all four variables are selected (*V = 4*), the estimated intercept $\beta _0$ is approximately *-1.67*. The final columns in Tables 7, 8 and 9 report the prior probabilities assigned to the states of each node for the initial construction of the network. The choice of uniform prior probabilities is a default setting and does not bias the final outcome. These values serve as a baseline to instantiate the Bayesian network: once the practitioner observes specific evidence in a real case, the node is instantiated and the probability of the observed state is updated to 1 (while the others are set to 0), thus triggering the probabilistic update across the entire model. A function node^5^ named Posterior_Suicide (represented by a double-border hexagon) has been included to allow the user to calculate the probability of suicide ($p_i$) based on the logistic regression parameters integrated within the network.

**Table 7:** Definition of nodes (observational evidence variables) depicted in Fig. 1, their states and associated probabilities

Node	Definition	States	Probability
P	Variable	*Home*	1/3
		*Indoor*	1/3
		*Outdoor*	1/3
WP	Variable	*Away from the body*	0.5
		*Near the body*	0.5
S	Variable	*No*	0.5
		*Yes*	0.5
Pc	Variable	*No*	0.5
		*Yes*	0.5

**Table 8:** Definition of nodes (regression coefficients) depicted in Fig. 1, their states and associated probabilities. Regression coefficients are model-specific for each value of the parent node *V*

Node	Definition	States	States of	Probability
			parent’s nodes	
V	Number of variables	1,2,3,4		0.25
B0	Regression coefficient	0.4700036	*V = 1*	1
		-1.9706681	*V=2*	1
		-1.1099319	*V=3*	1
		-1.6692052	*V=4*	1
B1	Regression coefficient	0.7086514	*V = 1*	1
		1.1185854	*V=2*	1
		0.9575097	*V=3*	1
		1.3930602	*V=4*	1
B2	Regression coefficient	-2.2046053	*V = 1*	1
		-1.464349	*V=2*	1
		-1.677087	*V=3*	1
		-20.046096	*V=4*	1
B3	Regression coefficient	0	*V = 1*	1
		3.141553	*V=2*	1
		2.722760	*V=3*	1
		2.834010	*V=4*	1
B4	Regression coefficient	0	*V = 1*	1
		0	*V=2*	1
		-19.1239	*V=3*	1
		-20.8047	*V=4*	1
B5	Regression coefficient	0	*V = 1*	1
		0	*V=2*	1
		0	*V=3*	1
		21.90186	*V=4*	1

**Table 9:** Definition of nodes (design matrix containing dummy variables used to represent qualitative variables in the regression model) depicted in Fig. 1 and their associated probabilities

Node	Definition	States	States of	Probability
			parent’s nodes	
X1	Variable	0	*Home*	1
		1	*Home*	0
		0	*Indoor*	0
		1	*Indoor*	1
		0	*Outdoor*	1
		1	*Outdoor*	0
X2	Variable	0	*Home*	1
		1	*Home*	0
		0	*Indoor*	1
		1	*Indoor*	0
		0	*Outdoor*	0
		1	*Outdoor*	1
X3	Variable	0	*Away*	1
		1	*Away*	0
		0	*Near*	0
		1	*Near*	1
X4	Variable	0	*No*	1
		1	*No*	0
		0	*Yes*	0
		1	*Yes*	1
X5	Variable	0	*No*	1
		1	*No*	0
		0	*Yes*	0
		1	*Yes*	1

## A decisional perspective: Bayesian decision networks

The logic of Bayesian decision networks can be effectively illustrated through the philosophical distinction between inference and decision. This distinction posits that inference and decision are intimately related, with inference serving as the starting point for decision-making [42]. Specifically, the act of identifying and considering a particular hypothesis as true is a decision that hinges on one’s strength of belief in that hypothesis (i.e., its posterior probability). The decision criterion also incorporates the losses, which quantify the relative undesirability of consequences arising from the combination of chosen decisions and actual hypotheses (i.e., the true states of nature).

As illustrated in Section “Bayesian decision framework”, the inferential task can be formulated as a decision problem, where $d_1$ represents the decision to report a questioned scenario as a suicide and $d_2$ represents the decision to report it as a homicide.

The formal Bayesian decision criterion in Eq. 4 involves calculating the expected loss for each decision and deciding in favor of $d_1$ whenever the expected loss associated with this decision ($\textrm{EL}(d_1)$) is smaller than the expected loss associated with the alternative decision $d_2$ (or, in other words, if the expected loss ratio is smaller than 1).

**Fig. 2:**
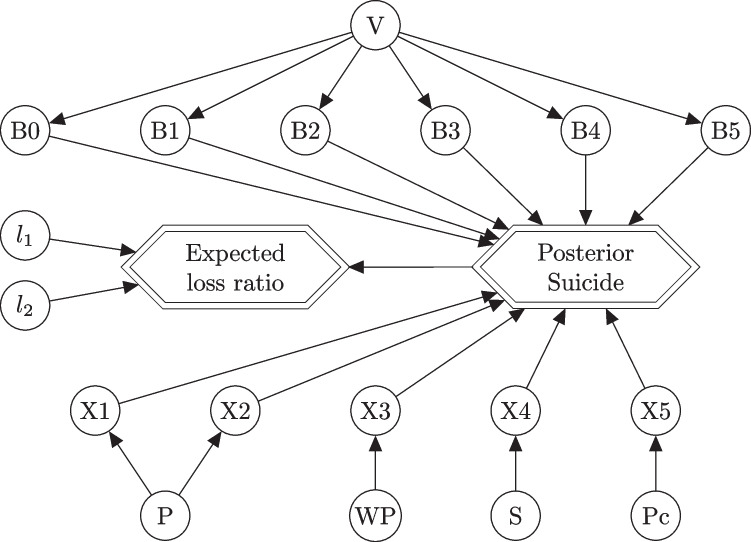
Bayesian decision network for inference and decision on the manner of death based on a set of observations collected at the crime scene

The Bayesian network shown in Fig. 1 can be extended to encompass the decision perspective^6^. Two numbered chance nodes, $l_1$ and $l_2$, have been added for shaping the loss function. An additional function node named Expected loss ratio (represented by a double-border hexagon) has been included to allow the user to identify the optimal decision according to the classification criteria in Eq. 4. This extended modelling formalism is presented in Fig. 2. The extra nodes are described in Table 10.

**Table 10:** Definition of nodes (loss values) depicted in Fig. 2 and their associated probabilities

Node	Definition	States	Probability
l1	loss incurred whenever decision $d_1$ is incorrect	*1,… ,10*	0.1
l2	loss incurred whenever decision $d_2$ is incorrect	*1,… ,10*	0.1

### Illustrative case: application of the Bayesian decision network

To demonstrate the practical implementation of the proposed model, let us consider the following scenario. Suppose a body is found inside a private residence. During the scene investigation, the weapon is discovered at a distance from the victim. Furthermore, the autopsy reveals no sharp force injuries on the back, and a review of the victim’s medical history shows no previous psychiatric comorbidities. We will now illustrate how this evidence is integrated into the proposed Bayesian Decision Network to support the decision-making process.

**Fig. 3:**
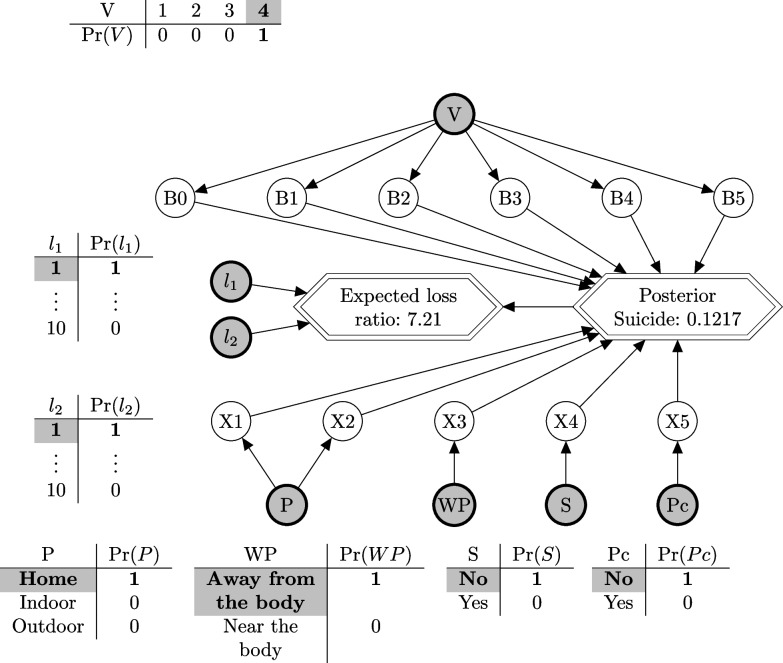
Extended representation of the BN in Fig. 2 for scenario n. H7. Instantiated nodes are indicated in bold over grey shaded area

First, the specific findings are entered as evidence (instantiated) into the respective nodes of the network. Figure 3 presents an application where the four variable nodes are instantiated for this scenario as follows:

- Place *->* *Home*
- Weapon place *->* *Away from the body*
- Sharp back *->* *No*
- Psychiatric comordibities *->* *No*

Note that *V* has been instantiated as *V=4* as all variables are available. A symmetric *0-1* loss function has been chosen, therefore nodes $l_1$ and $l_2$ have been instantiated as $l_1=1$ and $l_2=1$.

The node Posterior Suicide displays the posterior probability for the suicide hypothesis. The posterior probability for hypothesis $H_1$ (suicide) is equal to 0.1217, which implies a high probability (0.8783) for hypothesis $H_2$ (homicide). Since a *0-1* symmetric loss function is used (i.e., $l_1=l_2=1$), the expected loss ratio in Eq. 4 is equivalent to the posterior odds in favor of $H_2$ and is equal to 7.21,

9 $$\begin{aligned} { \frac{\textrm{EL}(d_1)}{\textrm{EL}(d_2)}=\frac{l_1}{l_2}\times \frac{\Pr (H_2\mid E,I)}{\Pr (H_1\mid E,I)} =\frac{0.8783}{0.1217}=7.21.} \end{aligned}$$

This result indicates that the expected loss associated with the decision to support a finding of suicide ($d_1$) is more than seven times higher than the expected loss of concluding in favor of homicide ($d_2$). Since the expected loss ratio is greater than 1, the Bayesian decision criterion in Eq. 4 identifies $d_2$ as the optimal choice, suggesting the classification of the manner of death as a homicide. Conversely, an expected loss ratio value smaller than 1 would point toward suicide as the optimal decision.

### Sensitivity analysis

The elicitation of an appropriate loss function can be a challenging task, since this choice directly impacts the optimality threshold (Eq. 4). Sensitivity analysis is therefore highly recommended to explore the impact of different choices on the final decision. To conduct a sensitivity analysis, we can consider various scenarios where different values of $l_1$ (i.e. the loss incurred whenever a case of violent death is incorrectly classified as a suicide) are taken into account. We will set $l_2$ (i.e. the loss incurred whenever a case of violent death is incorrectly classified as a homicide) equal to 1 and examine increasing values for $l_1$ (e.g., $l_1=1,2,...,10$). The rationale for choosing increasing values of $l_1$ is that wrongly deciding in favor of suicide is considered significantly more detrimental than the opposite. The optimality threshold will vary accordingly.

The logic underlying the sensitivity analysis can be clarified by rearranging the decision criterion in Eq. 4. Specifically, decision $d_1$ is preferred over decision $d_2$ when:

10 $$\begin{aligned} \frac{l_1}{l_2} < \frac{\Pr (H_1\mid E,I)}{1-\Pr (H_1\mid E,I)}. \end{aligned}$$

Rearranging terms, it is possible to find an optimality threshold representing the minimum posterior probability of suicide, $\Pr (H_1\mid E,I)$, required to justify the decision $d_1$. In particular, it can be shown that decision $d_1$ is preferred when the ratio of the losses is exceeded by the posterior odds:

11 $$\begin{aligned} \frac{l_1}{l_2} < \frac{\Pr (H_1\mid E,I)}{1-\Pr (H_1\mid E,I)}. \end{aligned}$$

Solving for $\Pr (H_1\mid E,I)$ yields the following optimality threshold:

12 $$\begin{aligned} \Pr (H_1\mid E,I) > \frac{l_1}{l_1+l_2}. \end{aligned}$$

Table 11 presents the optimal decision threshold corresponding to increasing values of $l_1$. Suppose, for illustrative purposes, that erroneously reporting a homicide as a suicide is considered ten times more detrimental than the reverse (i.e., $l_1 = 10$ and $l_2 = 1$). In such a case, the threshold in Eq. 12 is calculated as:

13 $$\begin{aligned} \Pr (H_1\mid E,I) > \frac{10}{10 + 1} = \frac{10}{11} \approx 0.91. \end{aligned}$$

This indicates that the probability of suicide must exceed 0.91 for decision $d_1$ to be considered optimal. As shown in Table 11, the ‘burden of proof’ required to support a finding of suicide increases significantly as the loss associated with a potential ‘false suicide’ (i.e., an undetected homicide) grows.

**Table 11:** Optimal decision threshold for increasing values of $l_1$

Loss value $l_1$	Threshold
1	0.50
2	0.66
3	0.75
4	0.80
5	0.83
6	0.86
7	0.87
8	0.89
9	0.90
10	0.91

## Discussion and conclusion

Deciding whether a case of violent death represents a homicide or a suicide is a fundamentally important step for the forensic medical discipline. The circumstances of death are not necessarily evident, and it is clear that erroneously classifying a homicide as a suicide will have a strong impact on investigative activities, posing a significant obstacle to the correct administration of justice.

Over the past 30 years, several relevant studies on sharp force fatalities have been published in the forensic literature, mostly retrospective, describing the frequency of specific features observed in sharp force homicides and suicides. From a forensic perspective, the aim has been to correlate the manner of death with both autopsy findings and complementary investigative information, including death scene examination data, relevant medical history, and post-mortem toxicological results obtained through additional investigative work. Despite the high number studies published, most of them include only suicides or homicides or present aggregated data from which specific feature frequencies cannot be extracted. Only a limited number of studies allow a direct comparison of the frequency of specific features across the two manners of death. The most relevant are the studies of Karlsson [5, 6] describing 105 suicides and 174 homicides from 1983 to 1992, representing the the largest numerosity. Lupi Manso et al. [9] reported data on 20 suicides and 57 homicides from 2012 to 2019. In the italian context we found two studies: Terranova et al. [7] with 20 suicides and 31 homicides from 1997 to 2019), and Vassalini et al. [8] with 28 suicides and 92 homicides from 1982 to 2012. In all these studies, some findigs have been almost unanimously correlated to a specific manner of death. Hesitation marks (antecubital fossa/wrists), a history of psychiatric disorder, and a weapon found near the body are frequent findings in suicides, while back injuries and clothing damage are more frequent in homicides [6–9]. Other variables remain controversial such as the victim’s age [3, 6], a high number of wounds [3, 43] as indicators supporting homicide. However, all the studies did not convert the specific features into probabilistic support for either hypothesis.

This article proposes a probabilistic approach to assign a probability value to the hypothesis of suicide or homicide, based on the information typically available to forensic pathologists. This approach offers potential utility both during the early investigative phase and in the subsequent evaluative stage. For this purpose, a Bayesian logistic regression model is proposed.

To enhance the reliability of this framework, the study’s dataset was carefully curated to include only clear-cut cases where the manner of death was unequivocally established, explicitly excluding any cases considered ambiguous. From a set of 33 available variables across 6 categories (see Table 2), four variables were selected to develop the model: the place where the body was found, the weapon’s position relative to the body, the presence of sharp-force injuries on the back, and the presence of psychiatric comorbidities. The first two variables are consistently recognized as discriminative in published studies [6, 9] , supporting homicide and suicide, respectively. “Weapon type"has been included in fewer studies and is often not discriminative probably due to a high proportion of “unknown” weapons, particularly in larger series [5, 6], and to the frequent use of kitchen knives in both suicides and homicides [7]. When reported, “Place where the body was found” variable supports that the victim’s home is more frequently observed in suicides [6, 9]. For this reason, we introduced home together with indoor and outdoor for this variable. The proposed model allows for obtaining probability values that in almost all cases point towards the correct manner of death (e.g. a probability greater (smaller) than 0.5 is generally obtained for cases scrutinized as suicide (homicide)).

A subsequent step performed in this article involved the extension of the proposed probabilistic approach to one encompassing a decision-making perspective. This extended model not only takes into account the probability of the manner of death for each case under examination, but also the undesirability of adverse outcomes associated with the decisional outcomes. The authors are well aware that implementing a decision-making approach in practice faces a significant obstacle: the concrete difficulty in eliciting the specific loss values that must be chosen to apply the model. Assigning numerical values to the undesirability of adverse outcomes can be challenging in real-world scenarios. In this respect, a sensitivity analysis was conducted with the aim of measuring the impact of different choices for these loss values on the recommended decision for cases of violent death. This helps to understand how robust the model’s indications are to variations in the assigned loss values.

It’s crucial to emphasize that the proposed decision-making approach, which is expected to be well-suited for the investigative phase of a violent death, does not refer to the final judgment on a case which falls under the purview of the judge. The main advantage of its implementation in the practice consists in highlighting the opportunity for more in-depth investigations or to broaden the scope of inquiries when there is strong doubt that a violent death case might be a homicide rather than a suicide. The practical implementation of the proposed probabilistic and decision-oriented approach can be facilitated by the use of probabilistic graphical models such as Bayesian networks.

In summary, adopting a probabilistic approach in forensic medicine is not merely an advanced methodological choice, but a fundamental requirement. It provides investigators and judicial authorities with a refined tool for managing the complexity of violent deaths, leading to a more transparent and rationally justified assessment of the discrimination task in sharp-force fatalities.

Despite the encouraging results, this study has limitations that must be acknowledged, including the small sample size of 51 cases (24 suicides and 27 homicides) and the retrospective nature of the data. The included cases were selected based on a clear-cut ground truth to allow for a rigorous initial validation of the Bayesian framework. While this is a necessary step for model building, it introduces a potential selection bias, as the model has not yet been tested on *borderline cases* where the manner of death is intrinsically ambiguous. Future research should focus on prospective validation using larger, multi-center datasets to further refine the model and its performance in real-world forensic practice.

In conclusion, this study represents a first attempt, based on existing literature and a case-related database, to adopt a Bayesian decision framework for the discrimination between suicide and homicide in sharp-force fatalities. Although based on a limited dataset of 51 cases, this work serves as a proof of principle demonstrating how probabilistic graphical models can effectively support forensic pathologists. The Bayesian network developed here allows for a transparent and reproducible integration of medical and circumstantial evidence, helping to quantify the value of each finding and minimizing the risk of intuitive bias in complex forensic evaluations. Clearly, it would be beneficial to have a greater number of cases available in future research.

While this model focuses on circumstantial and medico-legal data, it is intended to be part of a broader constellation of investigative information. Future research should explore the formal integration of these results with other types of evidence, such as digital or testimonial data, to provide an even more holistic support to the judicial decision-making process.

## Footnotes

1 Text available at: https://www.istat.it/wp-content/uploads/2024/11/Report_Vittime-di-omicidio_Anno-2023.pdf.

2 See, https://commonslibrary.parliament.uk/research-briefings/cbp-8224/ and https://www.ihemi.fr/sites/default/files/inline-files/GA_35_characteristics_homicides_paris.pdf.

3 The interested reader can refer to the seminal book of DV Lindley [21] for an extended presentation of Bayesian decision theory, [22] for a description of the role of decision theory in forensic science. Legal and forensic examples may be found, e.g., in [23–30].

4 The term ‘unconditional’ refers here only to the absence of explicit conditioning on other variables (nodes) in a network. Strictly speaking, a probability *\Pr (A)* should be considered conditional because there is always contextual knowledge, generally denoted by the letter *I*, which is used to associate a value to *\Pr (A)*. This implies that *\Pr (A)* should be written more correctly as $\Pr (A \mid I)$, though *I* is omitted to reduce notational burden.

5 A function node handles numerical functions within a Bayesian Network.

6 Forensic applications are discussed, e.g., in [26] and [41].
